# Effects of c*ofD* gene knock-out on the methanogenesis of *Methanobrevibacter ruminantium*

**DOI:** 10.1186/s13568-021-01236-2

**Published:** 2021-05-28

**Authors:** Jian Ma, Xueying Wang, Ting Zhou, Rui Hu, Huawei Zou, Zhisheng Wang, Cui Tan, Xiangfei Zhang, Quanhui Peng, Bai Xue, Lizhi Wang

**Affiliations:** Low Carbon Breeding Cattle and Safety Production University Key Laboratory of Sichuan Province, Animal Nutrition Institute, Sichuan Agricultural University, Chengdu, 611130 China

**Keywords:** *cofD* gene, Methane, Coenzyme F_420_, *Methanobrevibacter ruminantium*, Gene knock-out

## Abstract

This study aimed to investigate the effects of *cofD* gene knock-out on the synthesis of coenzyme F_420_ and production of methane in *Methanobrevibacter ruminantium* (*M. ruminantium*). The experiment successfully constructed a *cofD* gene knock-out *M. ruminantium* via homologous recombination technology. The results showed that the logarithmic phase of mutant *M. ruminantium* (12 h) was lower than the wild-type (24 h). The maximum biomass and specific growth rate of mutant *M. ruminantium* were significantly lower (*P* < 0.05) than those of wild-type, and the maximum biomass of mutant *M. ruminantium* was approximately half of the wild-type; meanwhile, the proliferation was reduced. The synthesis amount of coenzyme F_420_ of *M. ruminantium* was significantly decreased (*P* < 0.05) after the *cofD* gene knock-out. Moreover, the maximum amount of H_2_ consumed and CH_4_ produced by mutant were 14 and 2% of wild-type *M. ruminantium* respectively. In conclusion, *cofD* gene knock-out induced the decreased growth rate and reproductive ability of *M. ruminantium*. Subsequently, the synthesis of coenzyme F_420_ was decreased. Ultimately, the production capacity of CH_4_ in *M. ruminantium* was reduced. Our research provides evidence that *cofD* gene plays an indispensable role in the regulation of coenzyme F_420_ synthesis and CH_4_ production in *M. ruminantium*.

**Supplementary Information:**

The online version contains supplementary material available at 10.1186/s13568-021-01236-2.

## Introduction

The mitigation of greenhouse gases has become a hot topic in recent years owing to their severe environmental impact. Methane (CH_4_) is a strong greenhouse gas, accounting for approximately 16% of the total global greenhouse gas emissions (calculated as CO_2_ equivalent). Previously, several studies have been conducted to investigate the reduction of CH_4_ emissions to attenuate the greenhouse effect (André-Denis et al. 2011; Huang et al. 2020). In animal husbandry production, ruminants, contributing to 33% of the total CH_4_ emissions of human activities, are important sources of CH_4_ emissions. On the other hand, approximately 2 ~ 12% of the gross energy from feed can be transformed into CH_4_ during the ruminal fermentation of ruminants and exhausted because of unavailability (Johnson et al. 1995). In recent years, various strategies have been researched to inhibit CH_4_ production in the rumen, such as artificial regulation of ruminal microbiota structure, using the biological inhibitors and vaccines, chemical inhibitors, and nutritional control measures. Unfortunately, the rumen micro-ecosystems can adapt and recover the original methane-generating level soon after administration (Hook et al. 2010; Martin et al. 2010). Therefore, external regulations can not steadily reduce the CH_4_ production and emission from ruminants in the long term.

*Methanobrevibacter* is the dominant archaea in the gastrointestinal tract of herbivorous animals (Cersosimo et al. 2015). In the rumen, the *Methanobrevibacter ruminantium* (*M. ruminantium*) has higher methanogenic activity and adaptability of environmental changes (Li et al. 2014). As a kind of hydrogenotrophic methanogen, the *M. ruminantium* can utilize H_2_ and CO_2_ for CH_4_ production (Danielsson et al. 2012; Benepal 2012). Coenzyme F_420_ is a key metabolic coenzyme in the process of energy metabolism of hydrogenotrophic methanogens and involved in the critical steps of CH_4_ generation from CO_2_ by hydrogen reduction (Eirich et al. 1978). The generation of phosphodiester F_420_-0 by condensation of L-lactoyl diphosphate guanosine (LPPG) and Fo is a critical process for coenzyme F_420_ activation (Graupner and White 2001). This reaction is catalyzed by 2-phospho-L-lactate transferase (known as CofD in archaea) (Choi et al. 2001; Graupner et al. 2002). Hence, CofD plays an essential role in the process of F_420_ biosynthesis. However, at present, few researches have addressed the question of how CofD enzyme affects the synthesis of coenzyme F_420_, thereby regulating the methanogenic activity and growth of methanogens.

In the current study, a *cofD* activity deficient *M. ruminantium* strain was constructed by knocking out *cofD* gene using homologous recombination of the tetracycline resistance gene into the *cofD* sequence in the chromosome. The polymerase chain reaction and western blotting were performed to verify the success of constructed *cofD* knock-out strain and disruption of *cofD* activity. With the purpose of new molecular target towards mitigation of CH_4_ emission, the effects of a knocking-out *cofD* gene on the growth and methanogenic activity of *M. ruminantium* were investigated in anaerobic culture.

## Materials and methods

### Bacterial strains and plasmids

The representative ruminant *Methanobrevibacter ruminantium* M1 (DSM1093) (this strain have been deposited in a publicly accessible culture collection belonging to the DSMZ) used in this study was obtained from the CSIRO Microbiology Laboratory (Australia) friendly. The plasmid pUC19, pEASY®-T1, and pBR322 cloning vector were purchased from JRDUN Biotechnogy Co. Ltd (Shanghai, China). The strain *E. coli* DH5α was used as a plasmid cloning host. The pUC18-*cofD*-*tet* vector were constructed in this study.

### Chemicals and media

All the chemicals were analytical grade. *M. ruminantium* strain was cultured in the following liquid medium: yeast extract, 0.2 g/L; peptone, 0.2 g/L; NaHCO_3_, 6 g/L; L-cysteine-HCl∙H_2_O, 0.35 g/L; mineral salt solution I, 0.05%; mineral salt solution II,0.05%; Balch trace element solution, 0.01%, and 0.1% resazurin (W/V) 0.001%, in which solutions were prepared previously and stored at 4 ℃. In our research, the modified liquid medium (BJ) was supplemented with 0.1% clarified ruminal fluid (the ruminal fluid was centrifuged at 4 °C and 13,000 r/min for 15 min, and the supernatant was collected). Finally, the deionized water was added to a total volume of 1 L. Mineral salt solution I (values in grams per liter): K_2_HPO_4_∙3H_2_O, 7.86; Mineral salt solution II: KH_2_PO_4_, 6; (NH_4_)SO_4_, 6; NaCl, 12; MgSO_4_, 1.2; MgSO_4_∙7H_2_O, 2.5; CaCl_2_, 1.2; CaCl_2_∙2H_2_O, 1.6; Balch trace element solution: nitro acetic acid, 1.5; MgSO_4_∙7H_2_O, 3.0; NaCl,1.0; MnSO_4_∙2H_2_O, 0.5; FeSO_4_∙7H_2_O, 0.1; CoCl_2_∙6H_2_O, 0.1; CaCl_2_∙2H_2_O, 0.1; ZnCl_2_, 0.1; CuSO_4_∙5H_2_O, 0.01; AlK(SO_4_)_2_, 0.01; H_3_BO_3_, 0.01; Na_2_MoO_4_∙2H_2_O, 0.01. The liquid medium pH was adjusted and maintained at 6.9 ~ 7.0. The medium was prepared under an 80% nitrogen and 20% carbon dioxide gas phase by the Hungate technique as modified Bryant and Robinson (Balch and Wolfe 1977). The aliquots of the medium were separated under strictly anaerobic condition via autoclaving at 124 ℃, and all roller tubes and vials were capped with rubber plugs and aluminum caps. For *E. coli* incubation, a Luria–Bertani (LB) solid medium containing 10 g/L tryptone, 5 g/L yeast extract, and 10 g/L NaCl was used and agar was added to the medium to a final concentration of 2% for preparation of solid medium.

### Preparation of target gene *cofD* fragment

The *M. ruminantium* strain was inoculated in deoxygenated sterilized Hungatetubes contained with liquid medium for recovery and culture at 39 °C for 30 h. Genomic DNA was extracted from collected thalli cells using the Bacterial Genomic DNA Extraction Kit (Takara, Beijing, China). The *cofD* gene was amplified by PCR using primers *cofD*5’and *cofD*3’ (Table 1). Amplification conditions were set at 94 °C for 10 min followed by 30 cycles at 94 °C for 45 s, 60 °C for 45 s, 72 °C for 45 s, and 72 °C for 10 min. Amplified *cofD* gene segments were excised from agarose gel and purified by QIAquick PCR purification Kit (Qiagen, Hilden, Germany). The target gene *cofD* was ligated with the vector pEASY-T1 at a molar ratio of 3:1 at 37 °C for 15 min. The ligation products were transformed into *E. coli* DH5α strain and screened with Blue-White Screenings on the LB plates containing Amp, IPTG, and X-gal. The transformed bacteria were amplified in LB liquid medium (containing 0.1% ampicilin), 37 °C, 250 r/min shock culture for 2 h, then verified by colony PCR. Positive clones were sent to the BGI Company (Beijing Genomics Institute) for sequencing, and homology comparisons were performed on GeneBank.

**Table 1:** List of reference genes and primers

Reference genes	Sequence (5’ → 3’)	bp
*cofD*-F	CGGAATTCATGATAACCATCCTTTCCG	906
*cofD*-R	CGCTCGAGCAGTTCTAACACCACTTTTGC	
*tet*-F	CGCATCGATTAGTTCTCATGTTTGACAGCTTATCTTCGAT	1288
*tet*-R	TTAATCGATTCAGGTCGAGGTGGCCCGGCT	

### Amplification of tetracycline resistance gene

Methanogens belong to the gram positive bacteria, and the tetracycline resistance gene can be expressed in gram positive bacteria according to its characteristics. Thus, in this experiment, it is selected as a marker gene for constructing a vector. The *Cla* I cleavage site was added to the two specific primers *tet*-F and *tet*-R of the tetracycline resistance gene (Table 1). The tetracycline resistance gene was specifically amplified using pBR322 plasmid DNA as a template by YEATaq polymerase (Takara, Beijing, China). The purified PCR product was ligated to the cloning vector pEASY-T1 at 37 °C for 15 min. The resulting plasmid was transformed into *E. coli* DH5α strain and screened with ampicillin. The extracted plasmid was named pEASY-T1-*cofD* and pEASY-T1-*tet* and sequenced.

### Construction of recombinant plasmid pUC18-*cofD*-*tet*

The constructed pEASY-T1-*cofD* recombinant plasmid and pUC18 plasmid vector were double digested with *Eco*RI and *Hin*dШ, respectively. After the pEASY-T1-*cofD* product was detected by gel electrophoresis, the *cofD* band was recovered and inserted into the pUC18 vector (double enzyme digested) to construct a pUC18-cofD recombinant plasmid. The constructed pEASY-T1-*tet* and pUC18-*cofD* plasmid DNAs were cleaved by *Cla* I respectively. After the detection of pEASY-T1-*tet* by electrophoresis, the *tet* band was recovered and inserted into the pUC18-*cofD* recombination vector, which was digested at 20 °C for 25 min. The resulting plasmid was transformed into *E. coli* DH5α strain and screened with Blue-White Screenings to select recombinant.

### Identification of *cofD* gene knock-out strains

The constructed pUC18-*cofD*-*tet* recombinant plasmid was transformed into the *M. ruminantium* by electroporation. The mutant strains were screened using tetracycline plates. After anaerobic culture, the knock-out and wild *M. ruminantium* bacterial fluid were collected and the DNA and RNA were extracted. The *cofD* gene expression and the CofD enzyme protein expression were determined by qRT-PCR and western blotting. The housekeeping gene glyceraldehyde-3-phosphate dehydrogenase (GAPDH) was used as a normalizing control.

### Comparison of *cofD* gene knock-out and wild-type strains

The mutant and wild-type *M. ruminantium* were anaerobically inoculated into Hungate tubes which equipped with optimized liquid medium. The inoculation amount of growth strains was 0.6 mL for each serum bottle and cultured at different time points (12, 24, 36, 48, 96 h) with three replicates at each time point. After inoculation, 40 kpa of CO_2_ was added to the Hungate tube, H_2_ was added to a pressure of 200 kpa, and the plate was placed in a constant-temperature incubator with a horizontal swing (40 rpm/min). The growth curve of *M. ruminantium* was measured using a spectrometer at a wavelength of 600 nm. The gas composition was analyzed by gas chromatography, including the respective proportions of the H_2_, CO_2_, and CH_4_. The absorbance of coenzyme F_420_ was measured by a spectrometer at a wavelength of 420.

### Statistical analysis

The data of *cofD* knock-out and wild-type strains were analyzed using the independent sample t-test of the SPSS statistical software (Version 20.0 for Windows; SPSS, Chicago, USA). The results were expressed as the mean and standard error of the mean (SEM). A significance level was indicated at *P* < 0.05, and a trend was declared at 0.05 ≤ *P* < 0.10.

## Results

### Plasmid construction for knock-out *cofD* and pEASY-T1-*cofD* plasmid construction

An 906 bp *cofD* gene fragment was successfully amplified from chromosome DNA of *M. ruminantium* using primers *cofD* designed. Amplification results are shown in Additional file 1: Fig. S1. The target gene *cofD* was ligated with the vector pEASY-T1 and confirmed by colony PCR. Six white monoclonal colonies grown on LB plates were randomly selected for further verification. DNA fragment sizes were observed in DNA electrophoresis analysis shown in Additional file 1: Fig. S2. The PCR products of positive clones were sequenced and the resulting sequences were subjected to nucleic acid sequence alignment. The results showed that the fragments were overlap by 828 bp (Identities = 821/828) and the sequence similarity was 99% (Additional file 1: Fig. S3).

### Plasmid pUC18-*cofD*-*tet* construction

The pCU18-*cofD*-*tet* ligation product was cultured in LB solid medium containing tetracycline and then inoculated into tetracycline-containing LB liquid medium. The bacterial liquid was used as a template and the *tet*-specific primers were used to amplify *tet*. The results are shown in Additional file 1: Fig. S4. The 9 clones picked out showed clear bands between 1000 and 2000 bp. Compared with the PCR products of the *tet* gene, the sizes were identical, indicating that the *tet* and pUC18-*cofD* vectors were successfully constructed. The pUC18-*cofD*-*tet* recombinant plasmid DNA was extracted from the above bacteria solution by an alkaline lysis method, and the extracted plasmid DNA was double digested with *Eco*RI and *Hin*dIII. The results are shown in Additional file 1: Fig. S5.

### Verification of *cofD* gene knock-out

**Fig. 1:**
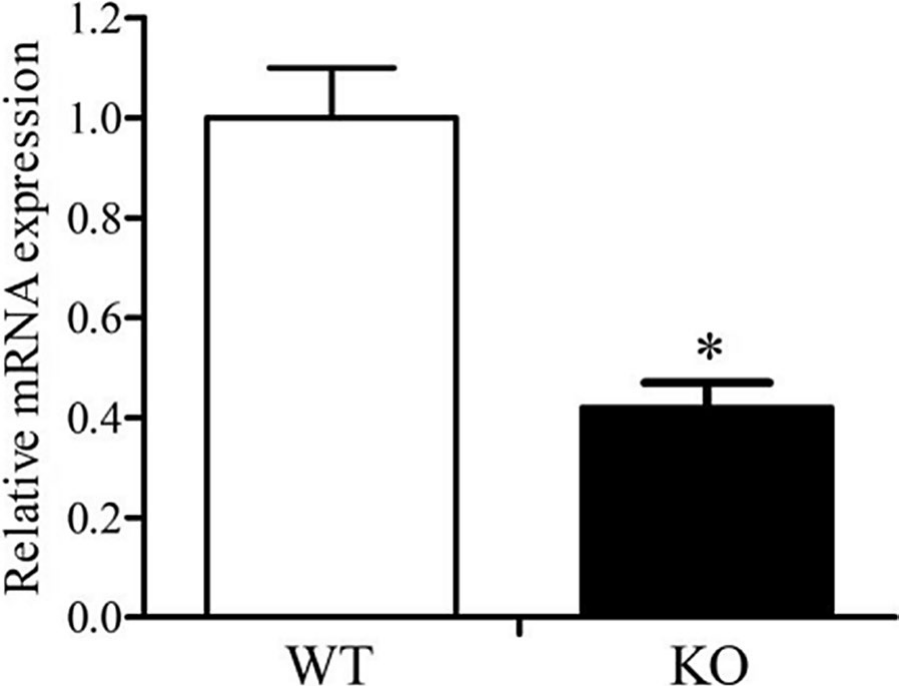
The differences of mRNA expression (mean and SD) of *cofD* in WT and KO *M. ruminantium*. Strains were incubated at 30° C for 72 h. *SD* standard deviation, *WT* wild-type, *KO* knock-out. The asterisk (*) indicates a significant difference (*P* < 0.05)

**Fig. 2:**
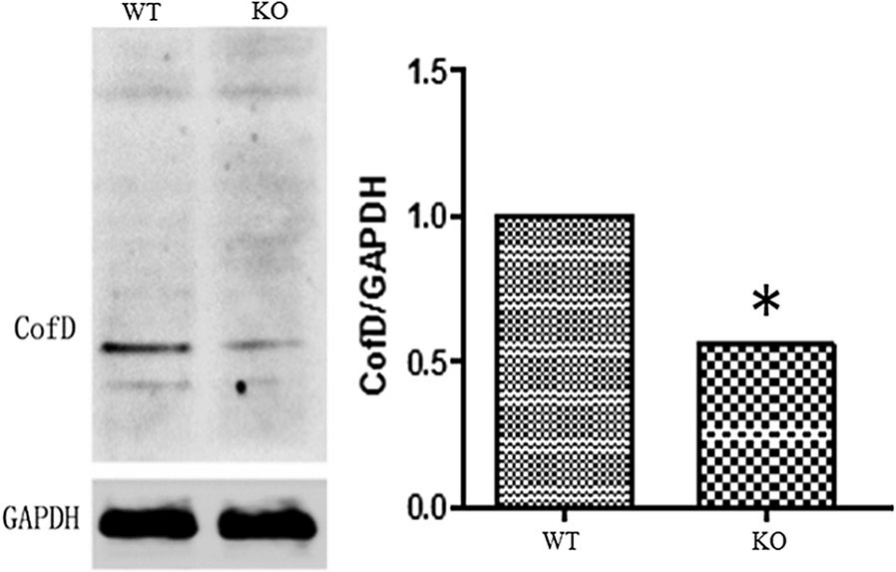
The differences protein expression (mean) of *cofD* in WT and KO *M. ruminantium*. Strains were incubated at 30° C for 72 h. *WT* wild-type, *KO* knock-out. The asterisk (*) indicates a significant difference (*P* < 0.05)

After the constructed pUC18-*cofD*-*tet* was transformed into the *M. ruminantium* by electrotransformation, the 100 μL of culture solution was coated on a tetracycline plate, and the mutant strain was selected by static culture at 30° C for 72 h. The expression level of *cofD* gene of mutant strain was lower (*P* < 0.05) than that of the wild-type strain (Fig. 1). Moreover, the expressing quantity of CofD enzyme protein in knock-out bacteria was significantly down-regulated (*P* < 0.05) as compared to the wild-type *M. ruminantium* (Fig. 2).

### Effects of *cofD* knock-out on the growth of *M. ruminantium*

**Fig. 3:**
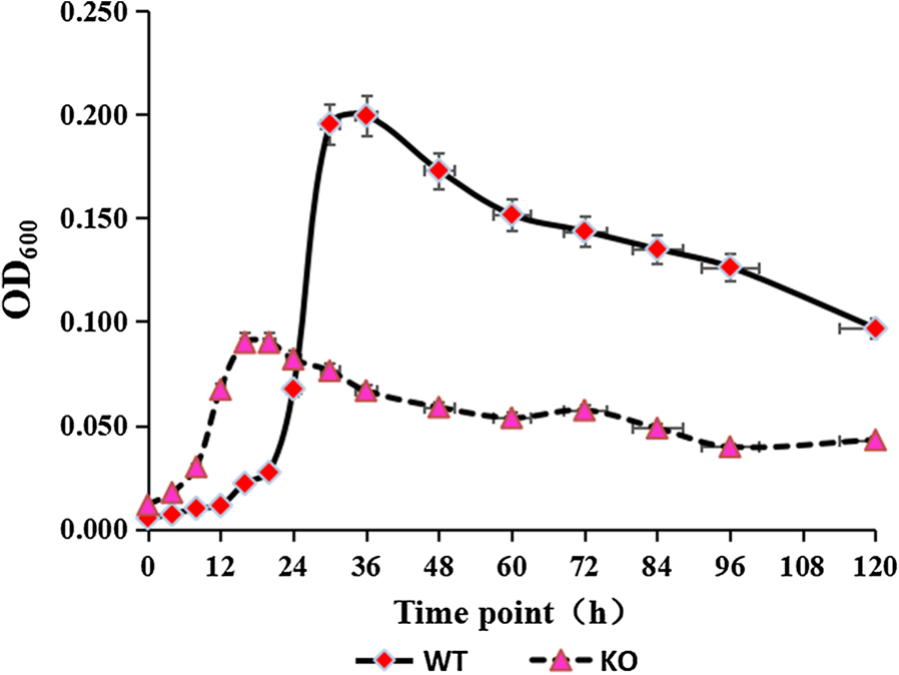
Growth kinetics of WT and *cofD* KO strains of *M. ruminantium* grown in BJ medium at 39 ℃ (n = 3). Growth was determined by measuring the absorbance of the cultures at 600 nm. *WT* wild-type, *KO* knock-out

The growth curves of wild-type and knock-out of *M. ruminantium* were respectively examined at 30 ℃ with equal inoculation amount (Fig. 3). After the *cofD* gene knock-out, the delay period of the mutant *M. ruminantium* was shorter than that of wild-type strains; besides, the *M. ruminantium* entered the logarithmic phase earlier. The logarithmic growth phase (12 h) of mutant *M. ruminantium* was lower in comparison with wild-type strains (24 h). As can be seen from Table 2, the maximal bacterial mass of *cofD* knock-out *M. ruminantium* was only approximately half of the wild-type strains, and the maximum specific growth rate was also lower (*P* < 0.05) than that of the wild-type strains.

**Table 2:** Comparison in the methane production and growth indexes of wild-type and mutant *M. ruminantium*

Indexes	Treatments		SEM	*P*-Value
	WT	KO		
The max amount of bacteria (OD value)	0.200	0.090	0.003	< 0.001
The maximum specific growth rate	0.017	0.008	0.001	< 0.001

*WT* wild-type, *KO* knock-out, *SEM* standard error of the mean

### Effects of ***cofD*** knock-out on the coenzyme F_420_ synthesis of ***M. ruminantium***

**Fig. 4:**
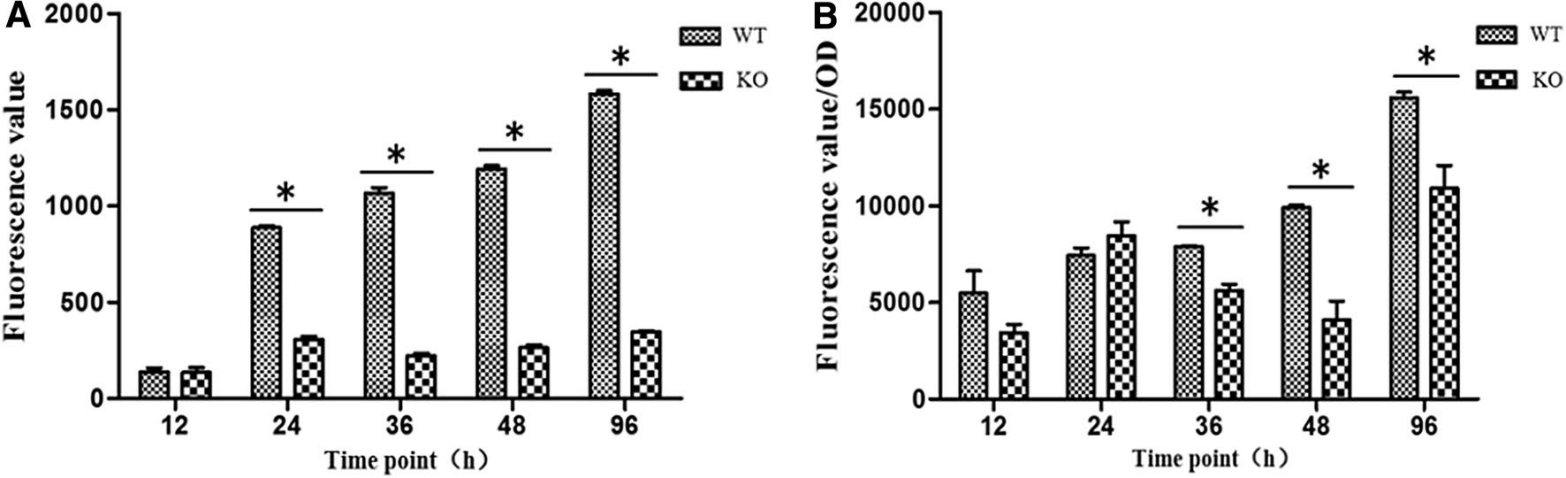
The content (mean and SD) of coenzyme F_420_ of WT and KO *M. ruminantium* in different culture time. **A** the content of coenzyme F_420_ of liquid; **B** the content of coenzyme F_420_ per OD_600_. *SD* standard deviation, *WT* wild-type, *KO* knock-out

As shown in Fig. 4, the fluorescence value of F_420_ was used to express the content of coenzyme F_420_ in this experiment. From the point view of growth of *M. ruminantium*, the coenzyme F_420_ content was increased gradually with the growth of the strain. From the total point of view (Fig. 4A), the content of coenzyme F_420_ in the *cofD* knock-out strains did not change significantly at 12 h (*P* > 0.05). However, at 24, 36, 48, and 96 h, the content of coenzyme F_420_ was significantly lower (*P* < 0.05) than that of wild-type *M. ruminantium*. Furthermore, the content of unit cell volume of coenzyme F_420_ between the *cofD* gene knock-out type and wild-type *M. ruminantium* at the same time point were analyzed (Fig. 4B). The content of coenzyme F_420_ per unit of biomass was decreased by 29%, 59%, and 30% at 36, 48, and 96 h respectively. All of them were significantly lower (*P* < 0.05) in knock-out than those in wild-type strains.

### Effects of *cofD* knock-out on the methane production of *M. ruminantium*

**Fig. 5:**
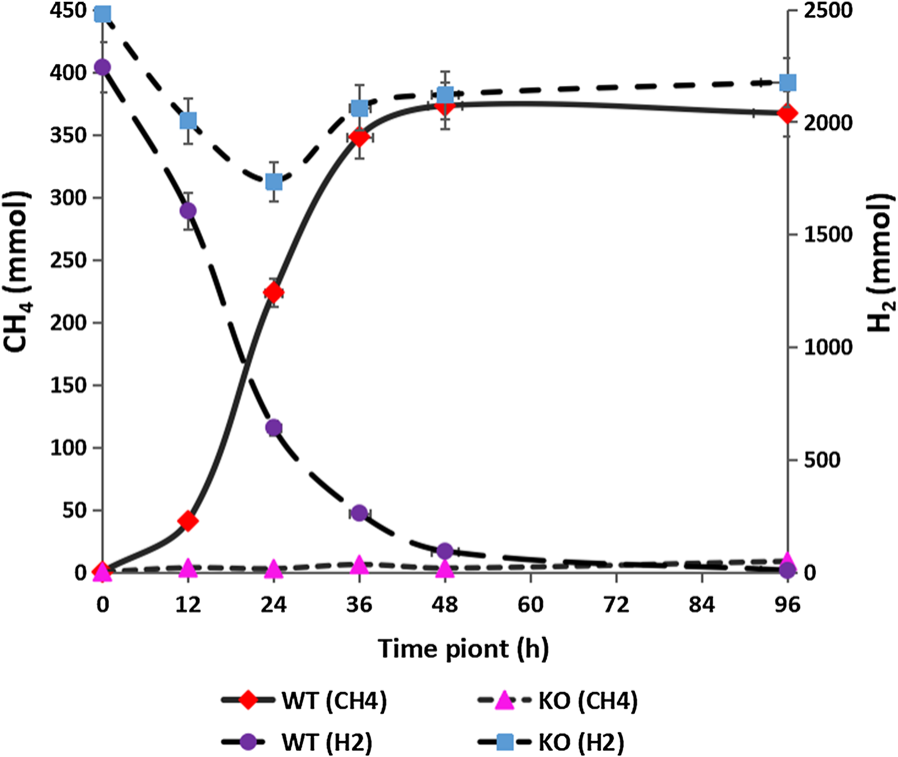
The concentration changes of methane production and hydrogen consumption in WT and KO *cofD* type *M. ruminantium* at 5 time points (12, 24, 36, 48, 96 h) (n = 3). *WT* wild-type, *KO* knock-out

From the CH_4_ production and H_2_ consumption curve of the wild-type *M. ruminantium*, it can be seen that as the incubation time increases, the concentration of H_2_ in the culture flask was continuously decreased, and the CH_4_ concentration was continuously increased (Fig. 5). The concentration of CH_4_ in the flasks reached a plateau at 48 h. Comparing with CH_4_ production and H_2_ consumption of wild-type *M. ruminantium*, it was found that the *cofD* knockout strains only consumed a markedly lower amount of H_2_ in the logarithmic growth phase to generate trace amounts of CH_4_, and the maximum consumption of H_2_ and the maximum production of CH_4_ for *cofD* knock-out strains were only 14% and 2%, respectively, of the wild-type strains.

## Discussion

In recent years, CH_4_ emissions from ruminants have caused not only adverse effects on the environment, but also loss of energy intake by animals. Therefore, regulation of methanogens in the rumen has become a research hotspot. A previous study has compared the 16S rRNA gene sequences of ruminal archaea derived from different ruminants and dietary compositions in 14 studies, and found that 92.3% of archaea in the rumen were originated from 3 genera, among which the *Methanobrevibacter* accounted for 61.6% and was the dominant methanogen in the gastrointestinal tracts of most herbivorous animals (Janssen and Kirs 2008).

As a strictly anaerobic bacteria, anaerobic condition is the key point to the successful cultivation of *M. ruminantium*. In the current experiment, the medium was boiled and deoxygenated. The anaerobic operation station was used for subpackage, inoculation, and incubation; besides, oxygen concentration was monitored in real-time using an oxygen controller to ensure strictly anaerobic conditions. The methanogens bacteria which is classified to *Methanobacillus* is mostly distributed in the two clades, including Smithii-gottschalkii-millerae-thaurei clusters and Ruminantium-olleyae clusters, and The *M. ruminantium* belongs to Ruminantium-olleyae clusters (St-Pierre et al. 2015). The *M. ruminantium*, *Methanobrevibacter thaueri*, *Methanobrevibacter smithii*, and *Methanosphaera stadtmanae* of the *Methanobacillus* have been confirmed to be the main methanogens in the bovine rumen (Jarvis et al. 2000; Zhou et al. 2009; Wright et al. 2007). Due to the short reproductive cycle of the fast-growing methanogens and residence time of ruminal chyme, it is more conducive to the establishment of fast-growing methanogens. In this study, the *M. ruminantium* entered a platform period at approximately 24 ~ 36 h. During the logarithmic growth phase, it consumed a large amount of H_2_, contributing to a lower level of hydrogen partial pressure in the rumen.

Methanal furans, tetrahydromethanopterin, coenzyme F_420_, coenzyme M, HS-Coenzyme B, and phenazine are closely related to the CH_4_ production of the methanogenic bacteria (Garcia et al. 2000). Among them, coenzyme F_420_ is a special low-potential electron carrier in high G + C gram-positive bacteria such as archaea and mycobacteria. In the process of CH_4_ production of methanogenic bacteria, coenzyme F_420_ is involved in the hydrogen transfer reaction as a two-electron transporter. Its oxidation–reduction potential is F_420_H_2_/F_420_ + 2^e−^(− 360 mV), lower than NAD(P)H/NAD(P) + 2^e−^(-320 mV) and FADH2 + FAD2^e−^(− 219 mV) (Purwantini and Mukhopadhyay 2009). The amino acid sequence of CofD enzyme is highly conserved among the archaea and bacterial organisms that produce coenzyme F_420_, but only weak sequence homologues appear in the thallus that do not produce coenzyme F_420_ (such as *Bacillus*) (Gorke et al. 2005). However, there are no other obligate anaerobic bacteria that contain coenzyme F_420_ and other substances that emit fluorescence at 480 nm and excitation wavelength is 420 nm. Therefore, fluorescence microscopy to detect the fluorescence produced by colonies has become an important technique for the identification of methanogenic bacteria.

Gene knock-out is an essential biological method for studying gene function and has been widely used. Sendai et al. (2006) constructed a gene knock-out model of α-1,3-galactosyltransferase on cattle by Cre/loxP method, which laid a foundation for application of multiple gene knock-outs in agricultural animals. Manabe et al. (2009) found that homologous knock-out of rinderpest virus could prevent bovine spontaneous prion encephalopathy and reduce the risk of bovine spontaneous encephalopathy. At the same time, study has also demonstrated that gene knock-out can block the formation of byproducts in microorganisms, thereby improving the yield and purity of the target product (Kim and Timmusk 2013). Gene knock-out provides new idea for biological research. However, few reports were carried out on the application of gene knock-out technology in the methane-related studies. In this research, homologous recombination technology was successfully used to screen the mutant strain of *M. ruminantium* in which *cofD* gene was knocked out, and the knock-out efficiency of screening was verified by PCR and enzyme digestion. In the process of gene knock-out vector construction, the pEASY-T1 vector was selected as the cloning vector of the *cofD* gene. The pEASY-T1 vector contains the β-galactosidase gene (*lac*Z) and the ampicillin resistance gene. If an exogenous fragment is inserted into a multi-cloning site, the *lac*Z gene and β-galactosidase would be inactivated. Since white colonies were produced, blue-white screening of positive clones could be performed using Amp/IPTG/X-gal selection medium. The pUC18 was used as a plasmid backbone, and a tetracycline gene was used as a resistance gene to construct a knock-out vector for *cofD* gene. This vector that was transformed into the *M. ruminantium* can not be replicated but has the opportunity to recombine with homologous genes homologously and express the tetracycline resistance, which can be selected. In this study, the *cofD* knock-out strains were selected, and the expression of *cofD* gene and CofD enzyme were significantly lower than those of wild-type strains.

Consistent with Liu and Whitman (2008) study, the results of current research displayed that in the logarithmic growth phase of wild-type *M. ruminantium*, it consumed greater amount of H_2_, which benefited to maintain a relatively lower level of H_2_ partial pressure in the rumen. The growth of strains was slower after the *cofD* gene knock-out. Moreover, the reproductive ability of strains was significantly reduced and it entered the logarithmic growth phase earlier. After entering the logarithmic growth phase, its coenzyme F_420_ content was significantly reduced, and only a small amount of H_2_ was consumed to produce CH_4_. The F_420_ is utilized in methanogens growth to oxidize their substrates. Thus, when the substrate is H_2_, the H_2_ can be coupled to reduce F_420_ by reducing F_420_ dehydrogenase (Frh) (Vitt et al. 2014). With knock-out of *cofD*, the *M. ruminantium* cell's redox driveability is reduced and lost partial ability to use H_2_, which may lead to a reduction in redox reaction of coenzyme F_420_ pool. Coenzyme F_420_ exists commonly in methanogens and its concentration in hydrogenotrophic methanogenic strain ranges from 100 to 400 mg/kg (Dolfing and Mulder 1985). The content of coenzyme F_420_ is different in varying species of methanogens. In addition, coenzyme F_420_ has been proved to be significantly higher in hydrogenotrophic methanogens than that in acetate-producing methanogens (Zábranská et al. 1985). In the study of *M. machellae* ⊿Frh mutants, it was found that Frh was necessary for the growth of methanogens under H_2_/CO_2_ culture conditions (Kulkarni et al. 2009). The character of blue-green or green fluorescence under the 420 nm UV laser generated by coenzyme F_420_ and methyl thiophene compounds is used to identify the presence of methanogens. However, Dong et al. (2010) concluded that the fluorescence value of coenzyme F_420_ can be used as an index to measure the activity of methanogens when upflow anaerobic sludge blanket reactor is used to treat waste water in soybean production. Some other studies on anaerobic fermentation found that the changes in the content of coenzyme F_420_ can be used to determine the activity of methanogens (Dolfing and Mulder 1985; Wang et al. 2011). In the current study, the CofD enzyme expression that was the corresponding protein product of F_420_ was significantly reduced after knock-out of *cofD* gene. Meanwhile, the synthesis of methanogenic coenzyme F_420_ was significantly reduced, indicating that the knock-out of the *cofD* gene resulted in a slower reaction of catalyzing the condensation of LPPG and Fo to produce F_420_ (Graupner et al. 2002). The results further demonstrated that the CofD enzyme was a key enzyme for the biosynthesis of coenzyme F_420_.

In summary, our results showed that the growth and proliferation ability of *M. ruminantium* was decreased after *cofD* knock-out, subsequently, the production capacity of CH_4_ by utilizing H_2_ in the *M. ruminantium* was reduced. The *cofD* gene knock-out reduced the expression of CofD enzyme and the synthesis of coenzyme F_420_ in the *M. ruminantium*. Our result may provide new insights to clarify coenzyme F_420_ and *cofD* gene involved in the methanogenic mechanisms that can affect CH_4_ production by methanogen, helping to develop strategies to reduce CH_4_ emissions from ruminants.

## Supplementary Information

**Additional file 1**: **Fig. S1**. Amplication of the cofD gene of M. ruminantium by using *cofD* primers. **Fig. S2**. The recombinant plasmid pEASY-T1-*cofD* was amplified using *cofD*-F and *cofD*-R as primers, and the transformants were identified by PCR. **Fig. S3**. PCR product sequencing of positive clones and comparison of similarities of *cofD* by Blast. **Fig. S4**. Identification of 9 monoclonal recombinant plasmids pUCl8-*cofD*-*tet* selected by PCR using tet specific primers. **Fig. S5**. Double digestion of pUCl8-cofD-*tet* recombinant plasmid DNA using *Eco*RI and *Hin*dIII, detection by 1% agarose gel electrophoresis. (DOCX 437 KB)

## Data Availability

The data presented in this study are available on request from the corresponding author.
