# Supplementary material for: Effects of cofD gene knock-out on the methanogenesis of Methanobrevibacter ruminantium
Source: AMB Express. 2021 May 28;11:77. doi: 10.1186/s13568-021-01236-2 (PMC8163928; doi:10.1186/s13568-021-01236-2)
Supplement: Supplementary file 1 — Additional file 1: Fig. S1. Amplication of the cofD gene of M. ruminantium by using cofD primers. Fig. S2. The recombinant plasmid pEASY-T1-cofD was amplified using cofD-F and cofD-R as primers, and the transformants were identified by PCR. Fig. S3. PCR product sequencing of positive clones and comparison of similarities of cofD by Blast. Fig. S4. Identification of 9 monoclonal recombinant plasmids pUCl8-cofD-tet selected by PCR using tet specific primers. Fig. S5. Double digestion of pUCl8-cofD-tet recombinant plasmid DNA using EcoRI and HindIII, detection by 1% agarose gel electrophoresis. (DOCX 437 KB) [file 13568_2021_1236_MOESM1_ESM.docx]

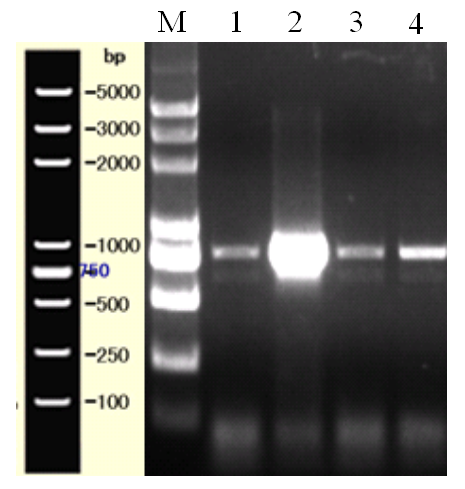


**Fig. S1** Amplication of the *cofD* gene of *M. ruminantium* by using *cofD* primers. Lane 1-4, all amplified fragments were between 750 and 1000 bp.


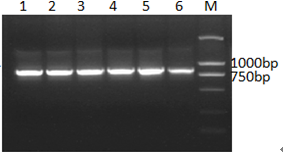


**Fig. S2** The recombinant plasmid pEASY-T1-*cofD* was amplified using *cofD*-F and *cofD*-R as primers, and the transformants were identified by PCR. Lane 1-6, positive transformants; M--DS2000Marker.


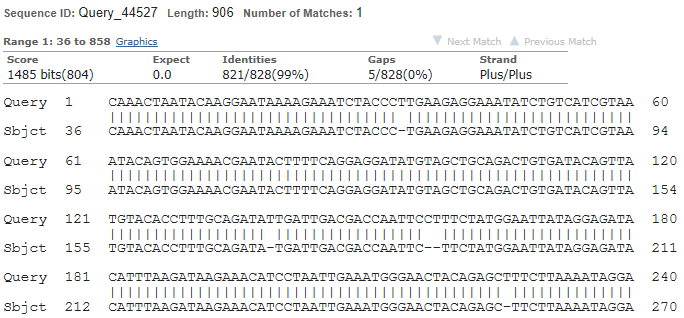


**Fig. S3** PCR product sequencing of positive clones and comparison of similarities of *cofD* by Blast.


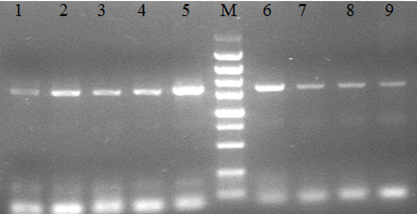


**Fig. S4** Identification of 9 monoclonal recombinant plasmids pUCl8-*cofD*-*tet* selected by PCR using *tet* specific primers. Lane 1-9, PCR products of *tet*; M, M-DS5000 Marker.


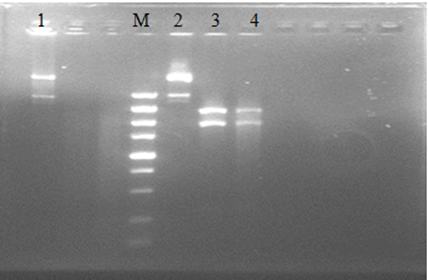


**Fig. S5** Double digestion of pUCl8-*cofD*-*tet* recombinant plasmid DNA using *Eco*RI and *Hin*dIII, detection by 1% agarose gel electrophoresis. Lane 1 and 2, *EcoR* I digestion; Lane 3 and 4, *Hind* III digestion; M, M-DS5000 Marker.
